# Effectiveness of Virtual Reality-Based Interventions for Managing Chronic Pain on Pain Reduction, Anxiety, Depression and Mood: A Systematic Review

**DOI:** 10.3390/healthcare10102047

**Published:** 2022-10-17

**Authors:** Ka Po Wong, Mimi Mun Yee Tse, Jing Qin

**Affiliations:** 1 Centre for Smart Health, School of Nursing, The Hong Kong Polytechnic University, 11 Yuk Choi Road, Hong Kong; 2 School of Nursing and Health Studies, Hong Kong Metropolitan University, 1 Sheung Shing Street, Ho Man Tin, Hong Kong

**Keywords:** chronic pain, virtual reality, phantom limb pain, chronic headache, chronic neck pain, chronic low-back pain, anxiety, depression, mood

## Abstract

(1) Background: Patients diagnosed with chronic pain suffer from long-term pain, which negatively affects their daily lives and mental health. Virtual reality (VR) technologies are considered a therapeutic tool to manage pain perception and mental health conditions. This systematic review aimed to appraise the efficacy of VR in improving pain intensity, anxiety, depression and mood among patients with chronic pain; (2) Methods: Five electronic databases were systematically searched using the terms representing VR and chronic pain. Quality assessment was conducted using Cochrane Collaboration’s tool and Newcastle-Ottawa scale; (3) Results: Seventeen peer-reviewed articles were included in this review. It was found that VR was able to reduce pain intensity in patients with phantom limb pain, chronic headache, chronic neck pain and chronic low-back pain. The effects of VR on the improvement of anxiety, depression and mood were not determined due to the inadequate amount of clinical evidence; (4) Conclusions: VR, especially immersive VR, improves pain outcomes and its effects may vary depending on the approach and study design. More research is still needed to investigate the clinical use of VR in patients with chronic pain.

## 1. Introduction

Chronic pain, one of the most common human experiences, is a complicated and troubling problem. According to the International Association for the Study of Pain’s definition, chronic pain is ‘pain that lasts or recurs for more than three to six months’ [1]. Up to 20% of people worldwide are affected by chronic pain at any given time field [2,3,4]. Common chronic pain types include headache, postsurgical pain, post-trauma pain, lower back pain, cancer pain, arthritis pain, neurogenic pain and psychogenic pain [5]. Chronic pain is a ubiquitous medical complaint that accounts for 15–20% of physician visits [6]. Acute pain typically resolves after tissue healing, but in certain individuals, it persists beyond normal healing time (i.e., between three to six months), contributing to chronic pain.

The situation of acute pain is entirely different from chronic pain [7,8,9]. Acute pain serves a protective purpose; it is evoked by stimuli, such as trauma, surgery, extreme temperature, pressure or illness, which injure or threaten to destroy tissues [10,11]. By contrast, chronic pain is not necessarily associated with physical traumatic events and lacks physiological warning function. The painful sensation cannot be simply explained based on nerve impulse processing in the somatosensory system. Similar to phantom limb pain (PLP), the patient experiences intense pain of the complete absence of neuronal input from an entire field of nociceptors [10]. Pain is always a subjective sense because the experience of chronic pain varies widely between people and even within an individual depending on the context and meaning of pain and the psychological state of the person [12].

Chronic pain is the leading cause of why patients seek medical care [2]. Inappropriate chronic pain management often results in reduced quality of life, alcohol and drug usage, physical dysfunction and mental disorders [13,14]. The relationship between depression and chronic pain exhibits bidirectional characteristics. Patients with a long history of pain disorders have an increased risk of depression and anxiety symptoms [15]. The significant relationship between suicidal thoughts and pain symptoms has also been well demonstrated. Suicidal ideation and attempts were more prevalent in people with chronic pain than in those without [16]. Individuals with chronic pain are two to three times more likely to commit suicide [17]. The World Health Organisation (WHO) has acknowledged chronic pain as an individual key risk factor for suicide [18]. Furthermore, recent evidence shows that chronic pain can lead to anatomical and functional alterations in the brain [19]. Chronic pain poses a huge financial burden to society in addition to physical and emotional burdens [20]. The yearly cost of chronic pain is about $635 billion in the United States, which is higher than the annual costs for cancer and heart diseases [21].

A range of medication options is available for the treatment of chronic pain, including the use of nonsteroidal anti-inflammatory drugs (NSAIDs), opioids, antidepressants and anticonvulsants [22]. However, two-thirds of patients with chronic pain living in the Grampian region of Scotland, UK reported dissatisfaction with pharmacological treatments [14]. Moreover, people using pain medication can develop an addiction and have physical dependence [23,24]. Given the limitations of conventional rehabilitation, developing new rehabilitation strategies for patients with chronic pain is imperative. The purposes of chronic pain rehabilitation include reducing pain levels, overcoming negative mental problems and reducing reliance on the use of pain medication. Continuing neurobiological discoveries have generated new ideas for the development of non-pharmacological rehabilitation, such as physiotherapy, psychological therapy and surgery, to treat pain. Simple psychological manipulation, such as attention distraction, can significantly reduce pain intensity because cognitive and emotional states have an enormous influence on pain perception.

Virtual reality (VR) is a potentially powerful tool for relieving pain by enhancing psychological well-being. VR can provide three-dimensional (3D) environments and multi-sensory stimulation to users. VR can produce therapeutically useful scenarios and allow its appropriate use [25]. VR draws much attention to the computer-generated world, leaving less cognitive capacity available to process pain singles. Recent research demonstrated that VR is a promising tool to help reduce pain among individuals undergoing medical procedures [26], urological endoscopies [27], physical therapy [28] and dental procedures [29]. A review conducted by Malloy and Milling [30] in 2010 evaluated the effects of VR distraction on relieving different types of pain. Kenney et al. [31] conducted a meta-analysis to examine the effectiveness of VR distraction in managing acute and chronic pain. A study by Scapin et al. [32] investigated the effects of VR in the treatment of burn patients. The review of Mallari et al. [33] in 2019 compared the effects of VR and non-VR treatments on acute and chronic pain reduction among adults. Another study conducted by Indovina et al. [34] assessed the use of VR for managing pain and distress during medical procedures. A review by Pittara [35] evaluated the effects of VR on pain management in cancer. A systematic review conducted by Wittkopf [36] examined the effectiveness of interactive VR in managing acute and chronic pain perception. Previous studies evaluating VR intervention for pain were limited by combining overall results of acute and chronic pain, investigation of certain types of pain only, lack of assessment of the effects of VR intervention on anxiety, depression and mood and short of covering patients in different age groups. To fill these research gaps, a systematic review on the efficacy of VR in dealing with different types of chronic pain for patients in different age groups and their anxiety, depression and mood is needed to determine the effect of VR-based pain management intervention on different types of chronic pain, anxiety, depression and mood.

An increasing number of studies have assessed the use of VR as an analgesic for patients with chronic pain. Considering the accumulating evidence, a comprehensive systematic review on the effectiveness of VR in managing pain perception and mental health conditions among patients diagnosed with chronic pain is needed. This review aimed to: (1) widely characterise empirical studies to date on the effectiveness of VR distraction in relieving different types of chronic pain, reducing anxiety and depression, and improving mood (2) identify the primary shortcomings of these studies, (3) provide clinical implications on adopting VR distraction in reducing chronic pain and (4) highlight research gaps and suggest avenues for future research directions. The findings of this review can assist therapists in VR design and selection for patients with different types of chronic pain and mental health conditions.

## 2. Materials and Methods

### 2.1. Search Procedure

This review was conducted using the Preferred Reporting Items for Systematic Review and Meta-Analyses (PRISMA) guideline [37]. This review was registered in the PROSPERO International Prospective Register of systematic reviews (registration number: CRD42022325706). Publications were searched in Medline, Embase, PsycInfo, CINAHL complete and Wanfang in December 2021. A search strategy that combined medical subject headings (MeSH) terms and keywords was developed to generate sets for the themes ‘chronic pain’ and ‘virtual reality’. Table 1 demonstrates our search strategy. Data within the 12-year range from January 2010 to December 2021 were retrieved, and collection was ended in December 2021. The reference lists of the selected studies were examined to identify those that may have been missed due to the limitation of the above-mentioned search terms.

### 2.2. Eligibility Criteria and Selection Process

Studies included in this review should meet the PICO principle (population/patients, intervention, comparison and outcomes) and the following criteria. The research design should be empirical studies, for instance, randomised control trials (RCTs), cross-sectional studies, case series, etc. This criterion was to ensure the comprehensiveness of the review. Studies should contain an investigation of patients with chronic pain diagnoses. The forms of VR interventions for managing chronic pain could be immersive and non-immersive. All comparators (e.g., treatment-as-usual, waitlist control, placebo group and no treatment group) were included. Studies should report the primary outcome obtained from any indication of pain intensity, and secondary outcome indicators responding to anxiety, depression and mood. There was no limit on the change in symptoms lasted for. Each session should be a minimum of 10 min which was applied in other pain reduction-related reviews (e.g., [38]). Other inclusion criteria were written in English, peer-reviewed and published after 2010. A systematic review was conducted by Malloy and Milling [30] in 2010 about the effects of VR distraction on chronic pain and afterwards, no summary work was conducted on the same topic. Therefore, to update the development of VR on chronic pain management in the recent decade, a systematic review including studies related to this issue after 2010 is necessary. The exclusion criteria were as follows: papers without empirical results, those focusing purely on theory and ethical issues and/or those with regulatory issues concerning the use of VR. Articles were screened in two stages. In the first stage, the title and abstract of all studies were screened and any study that appeared to measure the association between VR and chronic pain was held for further analysis. In the second stage, the full text was examined and accepted if the above-mentioned inclusion and exclusion criteria were met.

### 2.3. Data Extraction and Quality Assessment

The data items extracted from each selected study were evaluated using a modified Cochrane Collaboration’s tool for assessing the risk of bias for randomised controlled trials (RCTs) [39] and the Newcastle-Ottawa scale for nonrandomised studies (NRSs) [40]. The corresponding authors or the co-author of the studies with missing data were contacted for requesting the data. If the author had no response or the data were unavailable, then the studies were excluded from the analysis. The methodological quality and risk of bias of the included articles were assessed based on the established guidelines and quality-assessment tools [37]. Quality probes included research questions, recruitment strategies, randomisation, outcome evaluation and statistical analysis. Two reviewers evaluated all the articles independently. Discrepancies were discussed by three reviewers. Thirteen studies were further excluded from the systematic review due to non-pertinent outcomes. Two studies using duplicate data and two studies that were not available in English were also excluded, resulting in 17 studies included for data synthesis. The overall quality of the studies was assessed according to the criteria in Table 2.

### 2.4. Data Synthesis

A narrative synthesis approach was adopted to describe the research designs, participant characteristics, VR interventions, outcomes and findings in this systematic review. This is due to the heterogeneity of major data items (e.g., study designs, comparators, measures and outcomes).

## 3. Results

### 3.1. Study Selection

**Figure 1:**
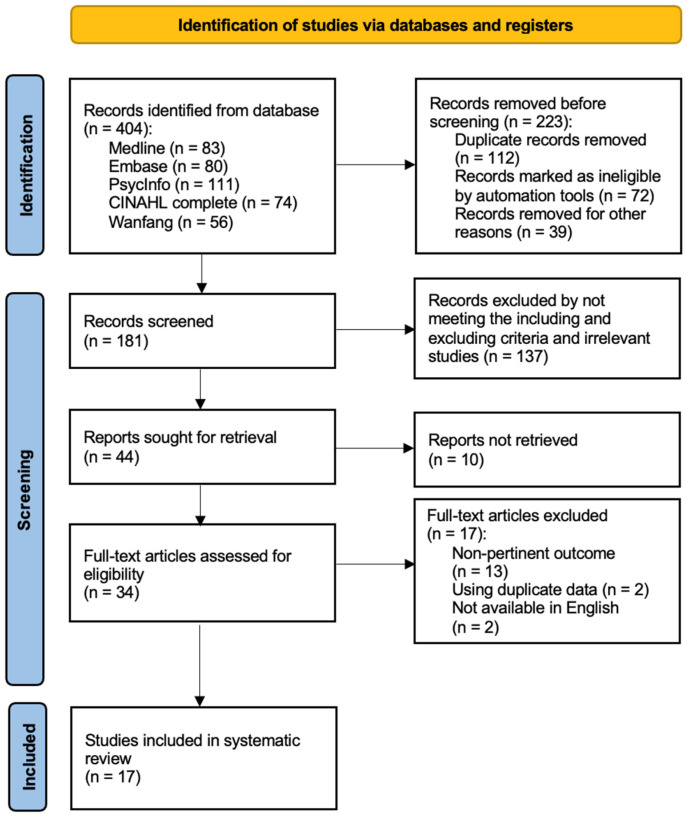
PRISMA flow chart of the article selection process.

The initial number of articles identified from databases was 404. After discarding duplicates and ineligible records, 181 papers remained. The full texts of the resulting 34 studies were retrieved and examined. After the full-text screening, 17 studies were excluded. The details of these excluded studies are shown in Supplementary Table S1 [41,42,43,44,45,46,47,48,49,50,51,52,53,54,55,56,57]. A total of 17 studies assessed VR-based interventions in chronic pain management. Further information on the selection process of the extracted studies is demonstrated in Figure 1.

### 3.2. Study Characteristics

Three studies were conducted in Canada, two each from the United States and Spain and one each in Australia, Belgium, Demark, Israel, Italy, Japan, Jorden, Slovenia, Sweden, Switzerland and Turkey. This systematic review included seven RCTs, seven quasi-experimental studies, one controlled study, one case series study and one before and after study. For the studies using RCT designs (*n* = 8), control groups consisted of treatment-as-usual, pain-relieving medication, virtual wheeling, laser training, traditional physical therapy, audio group, mindfulness-based stress reduction treatment and watching videos. Pretest and post-test assessments were used in all studies, with five studies having follow-up assessments ranging from 1 month to 6 months and one study having an assessment during the treatment. Ten studies were considered to have moderate quality, and nine studies were considered to have low quality. Detailed study characteristics are demonstrated in Table 3.

### 3.3. Participant Characteristics

A total of 605 participants were evaluated, and sample sizes ranged from 9 to 97 individuals. The participants were aged between 18 and 75. The participants were mainly female with a sample proportion of 58.5% (range from 26.3% to 100%). According to the categorisation of chronic pain types in the 11th version of the International Classification of Diseases (ICD-11) [72], five types of chronic pain were identified among the participants; these types include chronic primary pain, chronic cancer pain, chronic neuropathic pain, chronic headache and chronic musculoskeletal pain. Four studies did not indicate the types of chronic pain. The details of chronic pain categorisation are listed in Table 3. The participants were diagnosed by different approaches including the American College of Rheumatology, Numeric Rating Scale, Quantitative Sensory Testing, Neuropathic Pain Symptom Inventory, Short-Form McGill Pain Questionnaire, International Classification of Headache Disorders, Neck Disability Index, Defence and Veterans Pain Rating Scale and clinical evidence. Two studies [60,68] did not report the diagnosis method. Detailed participant characteristics are provided in Table 3.

### 3.4. Risk of Bias

Of the 17 included studies, eight of them were RCTs in which Cochrane Collaboration’s tool was used to assess the risk of bias of these RCTs. Five trials had low risk of random sequence generation. Four studies reported low risk of allocation concealment. The interventions in the trials may have unavoidable broken blinding. However, only three studies reported it and thus these three studies were judged to have low risk bias of blinding of participants and personnel. Only three trials, which mentioned blinding of outcome assessors, were judged to have low risk of bias of blinding of outcomes assessment. All trials had low risk of incomplete outcome data. Three studies had low risk of bias in selective reporting as the protocol, primary outcomes and secondary outcomes were reported in the studies. Only one study was judged to have low risk of other bias as the potential bias were reported in the study. The remaining nine studies were NRS, the methodological quality of which was assessed by the Newcastle-Ottawa scale. This scale had three main criteria, namely, the selection of the study groups, the comparability of the study groups, and the ascertainment of the outcome. The total scores of the studies ranged from 6 to 8. The scores of risk of bias of all studies are demonstrated in Table 3 and the details of risk of bias assessment are demonstrated in Supplementary Table S2.

### 3.5. VR Interventions

All studies examined a unique VR treatment and natural environment (Table 4). Ten studies adopted immersive VR technologies, and seven studies adopted non-immersive VR studies. The treatment purposes included rehabilitation (*n* = 7), pain reduction (*n* = 5), pain distraction (*n* = 3), activity management (*n* = 1) and relaxation (*n* = 1). The treatment ranged from 1 to 20 sessions, and the length of each session ranged from 1 min to 120 min. Two studies were designed with a home-based VR program and the remaining studies conducted the intervention in universities and hospitals. Different types of head-mounted displays (HMD) and motion sensors were used to facilitate the treatments for the participants.

### 3.6. User Engagement with the VR Interventions

The average attrition rate in the VR treatments across all studies was 11.7%, with a range of 0% to 53.3% (Table 4). The user engagement with the VR interventions was measured by Test of Playfulness, Global Perceived Effect, numeric rating scale and nonstandardised questions to indicate satisfaction, acceptability, enjoyment, motivation, attention and involvement. Eight studies did not report user engagement.

### 3.7. Pain Intensity, Anxiety, Depression and Mood Measurements and Outcomes

The details of the pain intensity measurements and the VR intervention outcomes in the included studies are summarised in Table 5. The measurements included Brief Pain Inventory (*n* = 3), Visual Analogue Scale (*n* = 5), numerical rating scale (*n* = 6), McGill Pain Questionnaire (*n* = 3), nonstandardised questions (*n* = 2), Short Leeds Assessment of Neuropathic Symptoms and Signs (*n* = 1) and Retroactive Pain Intensity (*n* = 1). Out of 17 studies, 13 studies reported a significant reduction in pain intensity after VR-based treatment.

Among the 17 studies, only seven studies assessed the mental health condition of the participants. The mental health condition consisted of depression, anxiety and relaxation. The measurements included Beck Depression Inventory (*n* = 2), patient health questionnaire (*n* = 1), State Anxiety Inventory (*n* = 1), numerical rating scale (*n* = 1) and nonstandardised questions (*n* = 3). Five studies reported a significant reduction in anxiety and depression and improvement in mood.

About 10 studies utilised immersive VR technologies, and eight of them reported a significant effect on pain reduction and three of them reported a significant effect on managing anxiety, depression and mood. Among the seven studies that adopted non-immersive VR, only four showed effectiveness in chronic pain reduction and two demonstrated effectiveness in mental health management. VR primary works through distraction to reduce the pain intensity. The details of the pain intensity, anxiety, depression and mood measurements and outcomes in the included studies are summarised in Table 5.

## 4. Discussion

This systematic review aimed to characterise empirical studies, describe the shortcomings of the selected studies, provide clinical implications of using VR distraction to manage chronic pain and mental health conditions, and highlight research gaps for future research directions. Despite particular methodological concerns that emerged across the studies, the review provides clinical evidence supporting the efficacy of VR distraction in chronic pain reduction, particularly in PLP, chronic headache, chronic neck pain and chronic low-back pain. The use of VR in pain reduction appears to be unpromising for patients suffering from chronic primary pain. The assessments of anxiety, depression and mood for patients with chronic pain were neglected in most studies, and thus the effectiveness of VR intervention for managing the mental health of patients with chronic pain was not concluded in this review. The study also suggested immersive VR potentially provided a way of exposing patients to a more attractive computer-generated environment that is more likely to exert an influence on pain reduction compared with non-immersive VR. Humans have a finite attentional capacity, and a distraction task consumes more portion of the capacities believed to leave less cognitive resources available for processing pain [12]. An immersive VR provides more sensory information that helps the person absent from the perception of pain.

### 4.1. Shortcomings of Included Studies

A significant problem is apparent with regard to the study methods used for estimating treatment effects. Firstly, most studies adopted a quasi-experimental design and RCT. The major concern of quasi-experimental studies is that randomisation is not applied, limiting the capability to draw a causal relationship between the intervention and the outcome. Meanwhile, conducting RCT can reduce confounding and bias. Secondly, the assessment of the mental health conditions of chronic pain after VR treatment was not conducted in most studies, confining our understanding of the feasibility of VR treatment in anxiety and depression reduction and mood management among patients with chronic pain. Thirdly, the hardware and software used in some studies were not clearly described. The commonly used tools for VR systems in these studies were HMD and digital computers. Several studies provided the content of the virtual environment without using software for developing the VR treatment. Fourthly, the arrangement of the duration of each session and the time interval between each session should be the significant factors of concern. According to the study of Strickland et al. [73], a 20 min duration is a threshold that normal adults tend to be discomforted with while using VR technologies. More than half of the selected studies conducted immersive VR and non-immersive VR sessions for more than 20 min. This effect possibly affects the results of the experiments and the physical health of the participants. Furthermore, a long-term interval may influence the actual effect of the VR treatment because treatments other than VR intervention on non-intervention days may generate effects that may enhance or weaken the effect of VR distraction. For example, Alemanno et al. [66] conducted the intervention twice per week in six weeks. During the non-intervention days, the patient may take other pharmacological or psychological treatments that may ultimately affect the outcome measures. Therefore, addressing the loophole on the influences in the non-intervention days is vital. Fifthly, some studies had a small sample size, for instance, the study of Garrett et al. [70] had nine subjects. Lastly, the quality of the included studies was moderate and low, implying that high-quality research and RCTs on this topic are still needed.

### 4.2. Clinical Implications

VR is needed to be applied more in daily clinical practice. Nonetheless, some clinical implications can be drawn at this stage. Five out of seven categories of chronic pain were included in this review. VR is used as a pain management tool in this clinical situation. VR technologies efficaciously reduce pain for patients with different types of chronic pain, particularly for adult patients. Immersive VR used as an adjuvant intervention is effective in relieving pain and anxiety for female patients with breast cancer and patients with chronic low-back pain. Non-immersive VR can improve motor function and neuropathic pain in patients with spinal cord injury-related pain, but the positive findings are limited to quasi-experimental studies. Immersive and non-immersive VR can restore phantom limb movement and alleviate PLP; however, an RCT is lacking to validate this significant finding. Non-immersive VR can reduce pain intensity and improve the quality of life among paediatric patients with chronic headache; these findings were limited to quasi-experimental studies. The pain intensity of patients with chronic low-back pain can be reduced under the VR intervention.

Each study initiated a cutting-edge VR treatment by using different hardware setups mainly including HMD and display screen and unique virtual environments created by VR software development tools. The hardware used in the studies are reasonably priced and commercially available in public markets. Active (e.g., walking in natural environments), passive (e.g., watching a video and movies) and interactive (e.g., playing interactive games) VR experiences were therapeutic for managing chronic pain among the patients. Rapid relief from pain perception and anxiety seem to be provided by the VR intervention. Out of two studies, one or two sessions varying from 10 to 20 min were adequate to prompt the relaxation of pain intensity and anxiety.

Adult patients with chronic pain reported high satisfaction and engagement with the VR treatment. This finding suggests that adults may find VR technologies as supportive and feasible in managing chronic pain; as such, adults may positively respond to digital technologies. Only one study examined the satisfaction of adolescent patients with the VR treatment and reported high satisfaction. This finding implies that experiments involving paediatric patients with chronic pain are scarce, and RCT is limited; thus, more RCTs are needed to be conducted for this group.

### 4.3. Recommendations for Future Research Directions

VR technology is a non-invasive tool used to treat pain. This review demonstrates the efficiency of this therapy in chronic pain management, anxiety and depression reduction and mood improvement. Further research regarding PLP and chronic headaches is still needed because current studies are mostly limited to quasi-experimental design. An RCT is also required to provide high-reliability findings. Among the 17 studies, the VR distraction programs in 15 studies were guided by therapists or instructors. Two studies were designed with a home-based VR program (i.e., [67,70]). Home-based VR programs can benefit patients with limited mobility and the elderly. However, without the guidance of therapists, the patients might not be able to attain the ideal effects of the rehabilitation program or injuries may incur. The advantages or disadvantages of home-based or self-administered VR intervention have rarely been discussed, and no comparison has been conducted on the effectiveness between guided and self-administrated VR treatment. These issues still need further research. Furthermore, participants in these studies were mainly adults and elderly but fewer children and adolescents. However, 20% to 35% of children and adolescents are affected by chronic pain [74]. Despite the significance of considering the paediatric population, few quantitative studies have investigated the efficacy of VR distraction in chronic pain reduction as well as mental health management. However, the use of VR also exerts side effects. The possible side effects and safety issues of VR treatment, which may vary from different forms of VR, have seldom been raised in discussion. This concern can be resolved using measurement tools, such as the Virtual Reality Sickness Questionnaire (VRSQ) [75], to evaluate the feelings of the users after conducting VR treatment. One of the significant issues that needed to be addressed is the duration of the treatment and the time interval of each session. As mentioned, a 20 min duration is an appropriate time for VR intervention, so researchers should design it with 20 min or less for each session. To avoid the effects of VR treatment interfered by other non-VR treatments, researchers can design an intensive program to evaluate the influence of the activities carried out on the non-intervention days during the period of the experiment or propose one session of VR treatment. These mentioned concerns are necessary to be resolved before VR becomes a normal daily clinical treatment for chronic pain reduction.

### 4.4. Limitations

This systematic review has several limitations. One of the purposes of this review is to identify studies on the use of VR for patients with chronic pain. The keywords related to VR in searching through electronic databases were not adequate to locate all relevant studies. To address this issue, the keywords used in the article-searching process were common naming. The second limitation of this review is that several study designs (i.e., RCT, quasi-experiment study and case study) were included. Although this approach can provide a comprehensive overview, the clinical evidence may be weakened. Furthermore, this systematic review only included the studies published after 2010 and thus a meta-analysis consisting of studies not limited to those published after 2010 is recommended to be conducted in the future. The last limitation is that only studies published in English language were included. Thus, non-English written studies related to this issue were neglected.

## 5. Conclusions

This review provides moderate evidence of the positive effect of VR treatment on reducing chronic pain and low evidence of the encouraging impact of VR intervention on anxiety and depression reduction and mood improvement. Most adult participants rated their VR experiment with high satisfaction; the attrition rates, however, were reported to have a large discrepancy. The effects may vary depending on the VR-intervention approach and study design. The promising results of VR, especially immersive VR, encourage its application as an adjunct therapy in clinical practices. Given the harmful effect of pharmacological treatment, many studies have proposed the use of VR distraction instead of the traditional method or as an adjunct analgesic technique. The main shortcomings observed in the studies include the inadequate number of RCT studies, a lack of evidence on moderate and long-term application, an inappropriate setting for the duration of treatment and time interval between each session and small sample size. Additionally, new areas can be explored, such as a comparison of the effects between self-administrated and guided VR treatment, an investigation of the paediatric population and an evaluation of the side effects of the VR approach. Due to the increasing application and continuous development of VR technologies among healthcare practitioners, more RCTs should be conducted to provide highly credible clinical evidence on the efficacy of VR distraction in patients with chronic pain.

## Figures and Tables

**Table 1:** Medical subject headings (MeSH) terms and keywords.

Theme	MeSH Terms	Keywords
Chronic pain	Chronic pain Arthralgia Back pain Cancer pain Metatarsalgia Musculoskeletal pain Neck pain Neuralgia Nociceptive pain	Persistent pain Chronic primary pain Chronic cancer pain Chronic posttraumatic and postsurgical pain Chronic neuropathic pain Chronic headache and orofacial pain Chronic visceral pain Chronic musculoskeletal pain
Virtual reality	Virtual reality	Virtual reality Virtual realities VR VR exposure Virtual environment

**Table 2:** Overall quality assessment using Cochrane’s tool for assessing risk of bias and Newcastle-Ottawa scale.

Quality Rating	Definition
High	RCTs with low risk of bias in all domains
Moderate	RCTs with high or unclear risk of bias in one or two domains NRSs with six to nine stars
Low	RCTs with high or unclear risk of bias in three or more domains NRSs with three to five stars
Very low	NRSs with three to five stars

NRS: nonrandomised study; RCT: randomised control trial.

**Table 3:** Description of study and participants characteristics.

Study	Country	Type of Pain	Diagnosis	*N*	Females, *n* (%)	Mean Age	Age Range	Study Design	Trial Arms	Measurements	Quality Score
Garcia-Palacios (2015) [55]	Spain	Chronic primary pain: Fibromyalgia	ACR	61	61 (100)	No mention	23–70	RCT	VRAM (31); TAU (30)	Pre, Post	4/7 (Low)
Mortensen (2015) [58]	Denmark	Chronic primary pain: Fibromyalgia	ACR	15	7 (100)	49.3	44–55	Quasi-experimental study	MCVG (15)	Pre, Post	6/9 (Moderate)
House (2016) [59]	The United States	Chronic cancer pain: Chronic pain post-cancer surgery	NRS	12	6 (100)	57.8	22–78	Quasi-experimental study	BrightArm Duo therapy (12)	Pre, Post, 8-week FU	7/9 (Moderate)
Mohammad (2018) [60]	Jordan	Chronic cancer pain: Breast cancer	No mention	80	80 (100)	52	30–70	RCT	VR (40); Morphine (40)	Pre, Post	3/7 (Low)
Jordan (2016) [61]	Spain	Chronic neuropathic pain: Spinal cord injury-related pain	QST	35	8 (29.1)	47.5	30–70	RCT	VWT (8); VW (7)	Pre, Post	3/7 (Low)
Villiger (2013) [62]	Switzerland	Chronic neuropathic pain: Spinal cord injury-related pain	Clinical evidence	14	5 (35.7)	52.7	28–71	Quasi-experimental study	VRAT (14)	Pre-pre, Pre, Post, FU at 12-16 weeks	8/9 (Moderate)
Ortiz-Catalan (2016) [63]	Sweden, Slovenia	Chronic neuropathic pain: PLP	Clinical evidence	14	No mention	50.3	28–74	Quasi-experimental study	VR (14)	Pre, Post, FU at 1, 3 and 6 months	7/9 (Moderate)
Osumi (2018) [28]	Japan	Chronic neuropathic pain: PLP	NPSI, SF-MPQ	19	5 (26.3)	49.1	23–71	Quasi-experimental study	VRR (19)	Pre, Post	6/9 (Moderate)
Shiri (2013) [64]	Israel	Chronic headache	ICHD	10	3 (30)	13.4	10.5–17.5	Quasi-experimental study	VR (10)	Pre, Post, FU at 1 and 3 months	7/9 (Moderate)
Sarig Bahat (2018) [51]	Australia	Chronic musculoskeletal pain: Chronic neck pain	NDI	90	63 (70)	48 (median)	18 or above	RCT	VR (30); Laser (30); Control (30)	Pre, Post, FU at 3 months	6/7 (Moderate)
Yelvar (2016) [65]	Turkey	Chronic musculoskeletal pain: Chronic low-back pain	Diagnosed by physicians	46	29 (63.0)	49.54	Less	RCT	VWT (23); Traditional Physiotherapy (23)	Pre, Post	4/7 (Low)
Alemanno (2019) [66]	Italy	Chronic musculoskeletal pain: Chronic low-back pain	Clinical evidence	20	11 (55)	47.5	19–72	Before-after studies	VRR (20)	Pre, Post	6/9 (Moderate)
Darnall (2020) [67]	The United States	Chronic musculoskeletal pain: Chronic nonmalignant low back pain or fibromyalgia	DVPRS	97	22 (29.7)	No mention	18–75	RCT	VR (47); Audio (50)	Pre, Mid, Post	4/7 (Low)
Wiederhold (2014) [68]	Belgium	Non-specific chronic pain: Average daily pain	No mention	40	No mention	No mention	22–68	Quasi-experimental study	VR (40)	Pre, Post	7/9 (Moderate)
Gromala (2015) [69]	Canada	Non-specific chronic pain	Clinical evidence	13	7 (53.8)	49	35–55	Controlled study	VR (7); Listen to the MBSR training audio track (6)	Pre, Post	1/7 (Low)
Garrett (2017) [70]	Canada	Non-specific chronic pain	Clinical evidence	9	6 (66.7)	45.3	31–71	Case series	VR (9)	Pre, Post	6/9 (Moderate)
Amin (2017) [71]	Canada	Non-specific chronic pain	Clinical evidence	30	13 (43.3)	No mention	22–29	RCT	Cardboard VR (10); VR (10); non-VR (10)	Pre, Post	2/7 (Low)

ACR: American College of Rheumatology; DVPRS: Defense and Veterans Pain Rating Scale; FU: Follow-up; ICHD: International Classification of Headache Disorders; Mid: During the treatment; MBSR: Mindfulness-based stress reduction; MCVG: Motion-Controlled Video Games; NDI: Neck Disability Index; NPSI: Neuropathic Pain Symptom Inventory; NRS: Numeric Pain Rating Scale; Pre: Pretest; Post-test; Pre-pre: Pre-pretest; Post: PLP: phantom limb pain; QST: Quantitative Sensory Testing; RCT: randomised control trial; SF-MPQ: Short-Form McGill Pain Questionnaire; TAU: treatment-as-usual; VR: Virtual reality; VRR: Virtual reality rehabilitation; VRAM: VRAT: Virtual Reality–Augmented Training; VR activity management; VW: Virtual wheeling; VWT: Virtual walking treatment.

**Table 4:** Details of the VR-based interventions and user engagement measure outcomes.

**Study**	**Types of VR**	Software	Purpose	Hardware	Length	Attrition (%)	Engagement Measures	Engagement Outcomes
Garcia-Palacios (2015) [55]	Non-immersive	EMMA, VR environment of a desert, a beach, a forest, a snowy landscape and a meadow	Activity management	Large screen, projector	6 2 h sessions in 3 weeks	1/31 (3.2)	NSQ	High satisfaction and acceptability
Mortensen (2015) [58]	Non-immersive	VR environment of 6 to 12 different activities (e.g., bowling, table tennis and volleyball)	Rehabilitation	Wii, PS3 Move, Xbox Kinect	15 30 min sessions	8/15 (53.3)	ToP	High enjoyment
House (2016) [59]	Non-immersive	Unity 3D, VR environment of nine games: Breakout 3D, Card Island, Remember that Card, Musical Drums, Xylophone, Pick & Place, Arm Slalom, Avalanche and Treasure Hunt.	Rehabilitation	Low-friction robotic rehabilitation table, computerized forearm supports, a display	16 20–50 min sessions in 8 weeks	6/12 (50)	NSQ	High acceptability
Mohammad (2018) [60]	Immersive	VR environment of deep-sea diving “Ocean Rift” and beach with the “Happy Place” track	Pain distraction	HMD with headphones	1 15 min session	0/40 (0)	No mention	No mention
Jordan (2016) [61]	Immersive	VR environment of an actor walking along a path	Pain reduction	No mention	1 20 min session	0/8 (0)	No mention	No mention
Villiger (2013) [62]	Non-immersive	Unity 3-dimensional (3D) game engine	Rehabilitation	3-degrees of freedom accelerometer sensor nodes, finger bend sensors	16–20 sessions in 4 weeks, 45-min/session	0/14 (0)	NRS	High enjoyment, motivation and attention
Ortiz-Catalan (2016) [63]	Non-immersive	Neuromotus™	Rehabilitation	Webcam, fiducial markersurface, electrodes over the stump	12 120 min sessions	0/14 (0)	No mention	No mention
Osumi (2018) [28]	Immersive	3D-CG, VR environment of mirror-reversed image	Rehabilitation	Oculus Rift HMD, Infrared sensor (Kinect for Winds v2)	1 20 min session	0/19 (0)	No mention	No mention
Shiri (2013) [64]	Non-immersive	ProComp Infiniti system	Relaxation	Electrodes	10 sessions	1/10 (10)	NRS	High satisfaction
Sarig Bahat (2018) [51]	Immersive	Unity-pro	Rehabilitation	Oculus Rift DK1 HMD equipped with 3D motion tracking	16 20 min sessions in 4 weeks	5/30 (16.7)	GPE satisfaction	High satisfaction (84.1%)
Yelvar (2016) [65]	Immersive	VR environment of a video clip was taken by a cameraman who was naturally walking down Ireland forest	Pain reduction, rehabilitation	iPod (Apple Inc., Cupertino, CA, USA) with video glasses (Wrap920)	10 15 min sessions in 2 weeks	1/23 (4.35)	NSQ: nonstandardised questions	Satisfied
Alemanno (2019) [66]	Non-immersive	An avatar reproducing online the performance of the patient who also gets an immediate visual and acoustic feedback on his/her performance	Rehabilitation	Computer workstation connected to a 6 degrees of freedom motion-tracking system (Polhemus G4), high-resolution LCD	12 60 min sessions over 4–6 weeks	0/20 (0)	No mention	No mention
Darnall (2020) [67]	Immersive	AppliedVR	Pain reduction	Oculus Go headset	4–8 sessions in 21 days, 1–15 min/session	12/47 (25.5)	NRS	High satisfaction
Wiederhold (2014) [68]	Immersive	VR environment of natural areas	Pain distraction	HMD	1 15 min session	6/40 (15)	No mention	No mention
Gromala (2015) [69]	Immersive	VR environment of a peaceful, non-distracting and safe environment	Pain reduction	DeepStream VR viewer	20-min	No mention	No mention	No mention
Garrett (2017) [70]	Immersive	VR environment of an Iceland, and a boat ride, 3D mandalas, an underwater, the solar system and a natural environment and active problem-solving experiences	Pain reduction	Oculus Rift DK2	30 min session in 1 month, 3 times a week	0/8 (0)	NSQ	No mention
Amin (2017) [71]	Immersive	Unity3D, Cryoblast	Pain distraction	Google LG Nexus 5 smartphone, Dodocase Virtual Reality Kit 1.2, Cardboard viewer with velcro, Oculus Rift Development Kit 2	2 10 min sessions in 1 day	0/10 (0)	NRS	High involvement

3D-CG: three-dimensional computer graphic; GPE: Global perceived effect; HMD: Head-mounted display; min: minutes; NRS: Numeric Rating Scale; NSQ: nonstandardised questions; ToP: Test of Playfulness.

**Table 5:** Details on pain intensity, anxiety, depression and mood measures and outcomes.

Study	Measures	Outcomes
Garcia-Palacios (2015) [55]	Pain intensity and interference: BPI Mood: BDI-II	No significant difference in pain intensity and depression in VRAM compared with TAU
Mortensen (2015) [58]	Pain improvement: VAS	No significant difference in pain improvement
House (2016) [59]	Pain intensity: NRS Depression: PHQ-9	No significant difference in pain reduction; large reduction in depression (8.3/10)
Mohammad (2018) [60]	Pain intensity: VAS Anxiety: SAI	Significant reduction in pain and anxiety in VR plus morphine compared with morphine alone
Jordan (2016) [61]	Pain intensity: NRS	No significant change in pain reduction; VWT is better than VW in pain reduction.
Villiger (2013) [62]	Pain intensity: NPS	Significant improvement in neuropathic pain
Ortiz-Catalan (2016) [63]	Pain intensity: NRS, MPQ	Significant improvement in PLP intensity
Osumi (2018) [28]	Pain intensity: NPS, SF-MPQ	Significant alleviation in PLP intensity
Shiri (2013) [64]	Pain severity: VAS	Significant reduction in pain severity
Sarig Bahat (2018) [51]	Pain intensity: VAS	Significant reduction in pain intensity
Yelvar (2016) [65]	Pain intensity: VAS	Significant improvement in pain intensity in VWT compared with traditional physiotherapy
Alemanno (2019) [66]	Pain intensity: MPQ, BPI; Mood: BDI	Significant improvement in pain intensity, mood and depression
Darnall (2020) [67]	Pain intensity: NRS Depression: NRS	Significant improvement in pain intensity and depression
Wiederhold (2014) [68]	Pain intensity: NRS	Significant reduction in pain intensity
Gromala (2015) [69]	Pain intensity: NRS	Significant reduction in pain intensity
Garrett (2017) [70]	Pain intensity: NSQ, BPI, S-LANSS Anxiety: NSQ, Relaxation:NSQ	Pain reduction during the VR among 62.5% of participants; no overall treatment difference in pain scores postexposure
Amin (2017) [71]	Pain intensity: RPI Anxiety: NSQ	Significant improvement in pain intensity in Cardboard VR (coupled with a smartphone) compared with traditional VR and significant improvement in anxiety

BDI-II: Beck Depression Inventory II; BPI: Brief Pain Inventory; MPQ: McGill Pain Questionnaire; NPS: Neuropathic Pain Scale; NRS: Numerical Rating Scale; NSQ: nonstandardised questions; PHQ: Patient Health Questionnaire; RPI: Retroactive Pain Intensity; SAI: State Anxiety Inventory; S-LANSS: Short Leeds Assessment of Neuropathic Symptoms and Signs; VAS: Visual Analogue Scale.

## Data Availability

All data utilized for the purpose of this study are available publicly and online.

## Supplementary Materials

The following supporting information can be downloaded at: https://www.mdpi.com/article/10.3390/healthcare10102047/s1, Table S1: Exclusion of full-text article (*n* = 17); Table S2: Risk of bias assessment.
