# Supplementary material for: Effectiveness of Virtual Reality-Based Interventions for Managing Chronic Pain on Pain Reduction, Anxiety, Depression and Mood: A Systematic Review
Source: Healthcare (Basel). 2022 Oct 17;10(10):2047. doi: 10.3390/healthcare10102047 (PMC9602273; doi:10.3390/healthcare10102047)
Supplement: Supplementary file 1 [file healthcare-10-02047-s001.zip › healthcare-1954378-supplementary.pdf]

Supplementary Table S1. Exclusion of full-text article (n = 17)

	Study	Reason	Category
1	Bermo et al. (2020) [41]	Pain caused by burn wound dressing and debridement, which was not considered as chronic pain.	Non-pertinent outcome
2	Chan et al. (2019) [42]	Pain caused by needle procedural pain was not considered as chronic pain.	Non-pertinent outcome
3	Dumoulin et al. (2019) [43]	Pain caused by needle-related procedures was not considered as chronic pain.	Non-pertinent outcome
4	Eijlers et al. (2019) [44]	Pain caused by surgery was not considered as chronic pain.	Non-pertinent outcome
5	Furness et al. (2019) [45]	Pain caused by wound dressings in burn care was not considered as chronic pain.	Non-pertinent outcome
6	Glennon et al. (2018) [46]	Pain caused by bone marrow aspiration and biopsy procedure was not considered as chronic pain.	Non-pertinent outcome
7	Gerçeker et al. (2021) [47]	Pain caused by Huber needle was not considered as chronic pain.	Non-pertinent outcome
8	Karaman et al. (2019) [48]	Pain caused by experimental ischemic pain created with a blood pressure instrument was not considered as chronic pain.	Non-pertinent outcome
9	McSherry et al. (2018) [49]	Pain caused by wound care procedures was not considered as chronic pain.	Non-pertinent outcome
10	Piskorz & Czub (2018) [50]	Pain caused by venipuncture was not considered as chronic pain.	Non-pertinent outcome
11	Semerci et al. (2021) [52]	Pain caused by venous port access was not considered as chronic pain.	Non-pertinent outcome
12	Soltani et al. (2018) [53]	The outcomes were about balance and joint flexibility after-burn.	Non-pertinent outcome
13	Walther-Larsen et al. (2019) [54]	Pain caused by venous cannulation was not considered as chronic pain.	Non-pertinent outcome
14	Díaz-García et al. (2015) [55]	The article is the same as the one included in the review (Garcia-Palacios et al., 2015)	Duplicated data
15	Sarig Bahat et al. (2018) [51]	The article is the same as the one included in the review Sarig Bahat et al., 2018)	Duplicated data
16	Wang & Li (2020) [56]	Only Chinese version	Not available in English
17	Song et al. (2018) [57]	Only Chinese version	Not available in English

Supplementary Table S2. Risk of bias assessment

	Garcia-Palacios, 2015	Mohammad, 2018	Jordan, 2016	Bahat, 2018	Yelvar, 2017	Darnall, 2020	Gromala, 2015	Amin, 2017
**Random sequence generation**	Low	Low	Unclear	Low	Unclear	Low	Unclear	Low
**Allocation concealment**	Unclear	Low	Unclear	Low	Low	Low	Unclear	Unclear
**Blinding of participants and personnel**	Low	Unclear	Unclear	Unclear	Low	Low	Unclear	Unclear
**Blinding of outcome assessment**	Unclear	Unclear	Low	Low	Low	Unclear	Unclear	Unclear
**Incompete outcome data**	Low	Low	Low	Low	Low	Low	Low	Low
**Selective reporting**	Low	Unclear	Low	Low	High	Unclear	High	Unclear
**Other bias**	Unclear	Unclear	Unclear	Low	Unclear	Unclear	High	Unclear

	Mortensen, 2015	House, 2016	Villiger, 2013	Ortiz-Catalan, 2016	Osumi, 2018	Shiri, 2013	Alemanno, 2019	Wiederhold, 2014	Garrett, 2017
**Selection**									
1	1	1	1	1	1	1	1	1	1
2	1	1	1	1	1	1	1	1	1
3	1	1	1	1	1	1	1	1	1
4	0	0	1	0	0	0	0	0	0
**Comparability**									
1	1	1	1	1	1	1	1	1	1
2	0	0	0	0	1	0	1	1	1
**Ascertainment**									
1	1	1	1	1	1	1	1	1	1
2	1	1	1	1	0	1	0	1	0
3	0	1	1	1	0	1	0	0	0
**Total**	6	7	8	7	6	7	6	7	6
